# Descemetocele and bilateral, severe *Pseudomonas* keratitis in an intensive care unit patient with Graves’ orbitopathy: A case report

**DOI:** 10.1097/MD.0000000000022481

**Published:** 2020-10-02

**Authors:** Yun Chen Hsieh, Chun-Chen Chen

**Affiliations:** a Department of Ophthalmology, Taipei City Hospital, Renai Branch; b School of Medicine, Taipei Medical University; c Institute of Clinical Medicine, National Yang-Ming University, Taipei, Taiwan.

**Keywords:** bacterial keratitis, eye care, Graves’ orbitopathy, intensive care unit

## Abstract

**Rationale:**

Exposure keratopathy (EK) is common among intensive care unit (ICU) patients, especially those under sedation and with incomplete eyelid closure. EK can be mild punctate epithelial erosions exhibiting spontaneous recovery; rarely, severe complications including bacterial ulcers causing corneal perforation or opacity could occur. We describe a patient with pre-existing Graves’ orbitopathy (GO) who developed bilateral, rapidly progressing bacterial keratitis due to EK with secondary aerosol inoculation from respiratory pathogens in ICU.

**Patient concerns:**

A 49-year-old intubated and sedated woman who underwent urgent craniotomy was admitted to ICU. The ophthalmology department was consulted for progressive chemosis. History of poorly controlled GO was revealed based on external ocular signs, including proptosis with lid retraction, and careful ophthalmological history taking. After 2 days of ICU admission, slit lamp examination revealed large inferior corneal epithelial defects and dellen (OU). Despite prescribing gentamicin ointment and lubricants, purulent discharge with corneal infiltration and thinning (OU) was observed 2 days later. Owing to a characteristic *Pseudomonas* odor from her endotracheal tube, corneal and endotracheal sputum cultures were obtained, which revealed *Pseudomonas aeruginosa* infection.

**Diagnosis:**

*Pseudomonas* keratitis secondary to EK

**Interventions:**

Topical fortified anti-*Pseudomonas* antibiotic eye drops with temporary tarsorrhaphy and lubricants

**Outcomes:**

Despite multiple treatments, she developed enlarging descemetocele in the left eye with severe corneal stromal destruction and severe visual impairment due to central corneal scar formation in the right eye. After 2 months, the descemetocele ruptured owing to generalized tonic–clonic seizures after cranioplasty. Therefore, she underwent urgent penetrating keratoplasty in the left eye.

**Lessons:**

GO increases ocular surface inflammation and exposure, which may exacerbate EK and subsequent complication risks. Careful monitoring and aggressive treatment through appropriate eye care regimen are required in these patients.

## 1 Introduction

Exposure keratopathy (EK), the most common ocular surface complication among intensive care unit (ICU) patients,[^1^,^11^] includes several severe complications such as bacterial ulcers that may cause corneal perforation or opacity and profound vision loss. In addition to a patient's ocular condition and length of hospitalization, general conditions including severity of systemic disease, level of consciousness, sedation duration, and metabolic derangements can add risks to impaired ocular protective mechanisms in critically ill patients, further leading to EK.[^2^,^3^,^4^,^5^] The incidence of EK in the ICU ranges from 6% to 57%, depending on the study method and eye care regimen.^[1]^ A small portion of these patients develop secondary infection; however, the true prevalence of bacterial keratitis in adult patients in the ICU remains unknown due to its low occurrence.

Graves’ orbitopathy (GO), an often-overlooked ocular condition in the ICU, may predispose patients to severe iatrogenic corneal injuries. Despite multiple treatments including lubrication, bandage contact lenses, temporary tarsorrhaphy, and various topical and systemic antibiotics, serious sequelae often incurred in eyes with deep corneal destruction. Clinicians should raise the awareness regarding the importance of ocular care to reduce the frequency of this avoidable condition.

## 2 Case History

**Figure 1:**
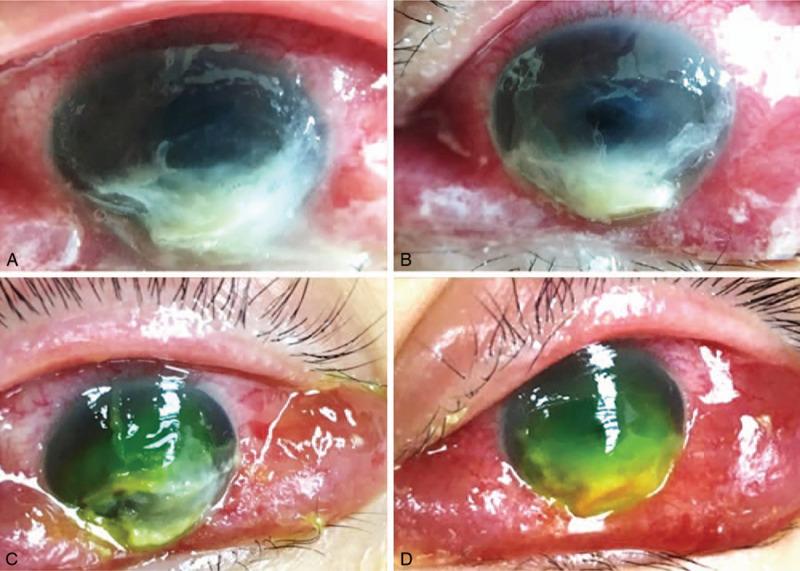
Bilateral corneal ulceration with a widespread corneal epithelial defect and inferior suppuration (A and C, Right eye; B and D, Left eye). Following the removal of the necrotic tissue, inferior corneal thinning and infiltration were observed 4 days after intensive care unit admission.

The ophthalmology service of Taipei City Hospital was contacted for a consultation regarding a 49-year-old female with a past history of hyperthyroidism who suffered from an acute right frontal lobe intracranial hemorrhage. The patient, still intubated and deeply sedated, was admitted to the ICU after an urgent craniotomy. Bilateral (OU) chemosis and incomplete eyelid closure were observed at ICU admission, and saline-soaked gauze was used to cover the eyes. In addition to profound proptosis with eyelid retraction and lagophthalmos in both eyes, a portable slit-lamp examination also demonstrated chemosis with conjunctival injection most prominent inferiorly, epithelial defects in the inferior third of the corneas, and dellen (OU). No conjunctival discharge or corneal infiltration was found at initial presentation. Detailed ophthalmology history revealed the patient was diagnosed with GO and had previously sought help from Chinese medicine and acupuncture practitioners. An endocrinology consultation was suggested to ascertain thyroid function. As mechanical eyelid taping failed due to severe chemosis, lubricants and prophylactic gentamicin ointment were prescribed. Despite this, purulent discharge with suppurative corneal infiltration and a shaggy corneal surface (OU) were found during ophthalmologic follow up within a few days. Corneal necrosis to half the stromal thickness with enlarging overlying epithelial excavation was demonstrated in both eyes with a fluorescein test (Fig. 1).

**Figure 2:**
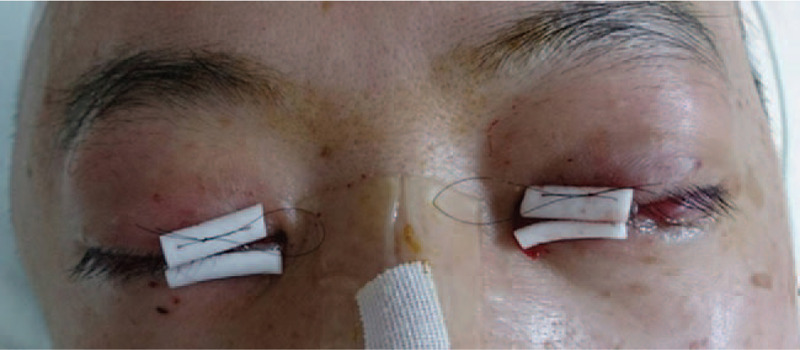
Temporary nasal tarsorrhaphy with silicone sponges and a double-armed nonabsorbable suture.

Because microbial keratitis was suspected, corneal specimens were obtained and subjected to Gram stain and culture. The patient was treated with broad-spectrum, fortified antibiotic eye drops including vancomycin (50 mg/mL) and ceftazidime (50 mg/mL). Due to a characteristic *Pseudomonas* odor from her endotracheal tube, an endotracheal sputum culture was also performed, and intravenous ceftazidime was administered. Corneal and sputum cultures both confirmed *Pseudomonas aeruginosa* infection. According to an antibiotic sensitivity test, topical antibiotics were shifted to piperacillin/tazobactam (50 mg/mL) and amikacin (10 mg/mL) with gentamicin ointment. Preservative-free artificial tears and duratear ointment were used for lubrication. Due to progressive corneal melting, a temporary lateral tarsorrhaphy (OU) was performed at the bedside to halt keratolysis (Fig. 2). Tarsorrhaphy stitches were removed 1 week later with signs of reepithelialization and stabilization of the stromal thinning. With lubrication and topical fortified anti-*Pseudomonas* antibiotic eye drops, the patient's ocular condition improved with reduced stromal infiltration and stromal edema.

**Figure 3:**
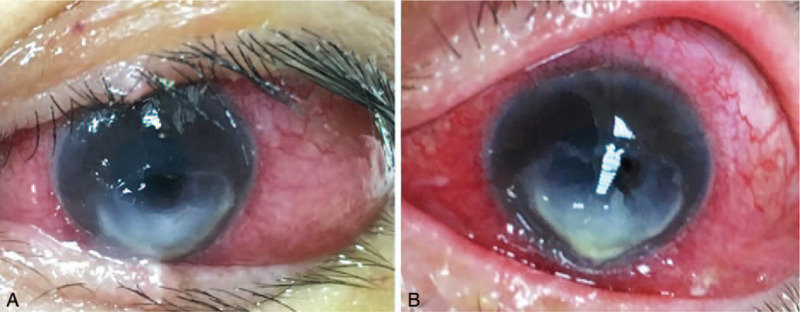
Corneal ulcer on the day of tarsorrhaphy removal (A) and 5 days later (B) demonstrated progression with increased infiltration and delayed reepithelialization (OS).

**Figure 4:**
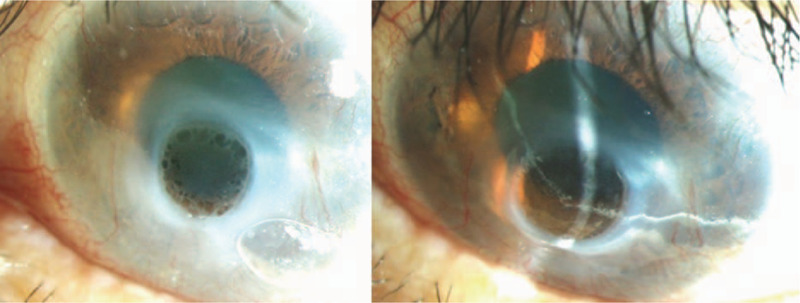
Enlarging descemetocele formation with bullous keratopathy (OS) subsequent to corneal stromal destruction.

**Figure 5:**
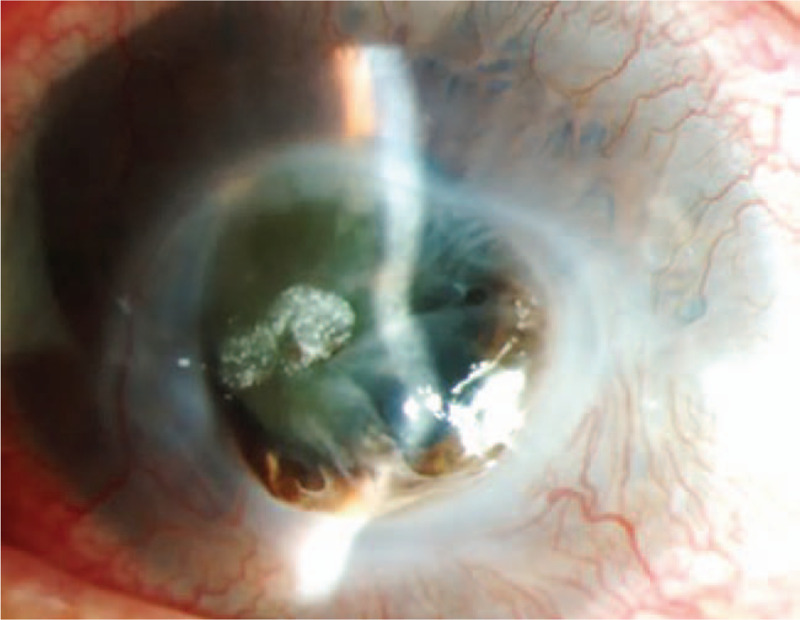
Descemetocele perforation with a flat and distorted anterior chamber and cataract formation (OS).

**Figure 6:**
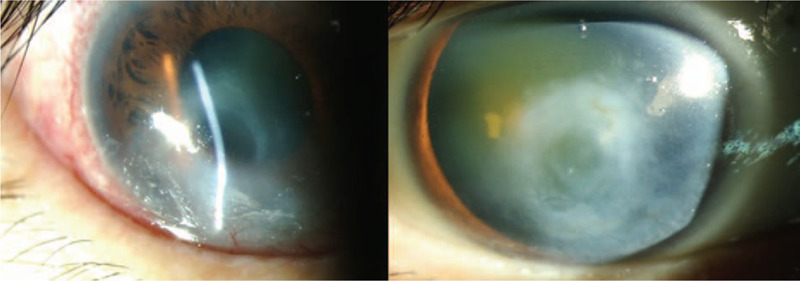
Corneal inferior thinning with progressive scar formation (OD).

After a few days, however, increasing necrotic tissue and discharge were observed (Fig. 3), and corneal and sputum recultures both revealed methicillin-sensitive *Staphylococcus aureus*. We thus added fortified vancomycin 50 (mg/mL) eye drops and applied aluminum eye shields with a sterile gauze coating (OU) to prevent further bacterial translocation. With the decrease in corneal infiltration, topical antibiotic eye drops were gradually tapered to reduce ocular surface toxicity, and bandage contact lenses and frequent preservative-free lubricants were applied. However, due to deep corneal ulcers and extensive stromal destruction, serious sequelae became inevitable in both eyes. In the right eye (OD), with more stromal preservation, the inferior corneal protrusion was less severe. In the left eye (OS), descemetocele with recurrent bullous epitheliopathy formed despite the use of a bandage contact lens with lubricants and systemic doxycycline treatment (Fig. 4). Unfortunately, generalized tonic-clonic seizures occurred after a cranioplasty 2 months later, and an ophthalmologic consultation then revealed ruptured descemetocele with a flattened and distorted anterior chamber structure (OS), indicating a left corneal perforation (Fig. 5). The patient underwent urgent penetrating keratoplasty (OS). After surgery, the best corrected visual acuity was 0.05 in the left eye and 0.1 in the right eye due to progressive corneal scar and cataract formation (Fig. 6).

## 3 Discussion

Strong evidence suggests that lagophthalmos is one of the major factors for EK in the ICU.[^7^,^9^,^10^] Critically ill patients frequently have lagophthalmos from sedatives or neuromuscular blockers used for neural system protection and to benefit other aspects of intensive care. These sedatives inhibit contraction of the ocular protractor muscles, block Bell's phenomenon and increase cornea exposure. More importantly, a comatose state or intubation obviates the patient's ability to covey pain and reflexive tearing associated with corneal injury, which often leads to late detection. In our patient with GO, exophthalmos with eyelid retraction and chemosis further accelerated ocular surface exposure and corneal epithelial breakdown. In critically ill patients, physiologic stresses may also induce GO exacerbation and further complicate the ocular condition.^[8]^

It has been well established that eye care protocols are effective in reducing ocular surface abnormality in the ICU.[^1^,^5^,^7^,^13^,^19^] In a large prospective study involving 301 ICU patients, Kuruvilla et al^[5]^ reported a low EK rate of 13.2% with routine eye care. In the protocol, an ophthalmologist performed bedside assessment using a portable slit-lamp, and no eye developed bacterial keratitis. As consulting an ophthalmologist for a daily ocular survey may be impractical, McHugh et al^[12]^ demonstrated the effectiveness of ocular screening by intensive care staff. With fluorescein, a blue filter pen light, and proper training, intensivists can safely detect corneal erosion with reasonable sensitivity and specificity.

It is uncommon for bacterial keratitis to occur without risk factors in hospitalized patients, and EK with the disruption of natural ocular defenses are major precursors of bacterial keratitis. With lacrimal dysfunction and epithelium break down, infection from exposed environmental pathogens may occur.^[6]^ In the unique environment of the ICU, most ocular infections are preceded by colonization of respiratory tract pathogens.^[18]^ In a study comprising 13 cases in 9 ICU patients with bacterial keratitis, Parkin et al^[14]^ highlighted *Pseudomonas* as the leading cause of devastating keratitis often requiring further surgical repair. More than half of these *Pseudomonas*-related keratitis cases affected the eyes bilaterally, severely impacting patient quality of life.

Evidence has shown that the incidence of pathogen translocation onto the ocular surface is time-dependent, and the risk rises with mechanical ventilation. Mela et al^[18]^ reported that the mean duration of ICU stay in sedated and ventilated patients with one species of abnormal conjunctival bacterial colonization was 26 days, and it was 42 days in those with 2 different species. By contrast, our patient had a rapidly progressive course. Besides early-onset chemosis and ocular surface injection during brain surgery, she experienced large corneal epithelial defects with dellen only 2 days after ICU admission and *Pseudomonas* keratitis 4 days after. She also had a second bacterial colonization after 15 days in the unit. Besides her other various risks, we believe that her concurrent thyroid eye disease is one of the contributing factors for this. Most patients with GO have dry eye syndrome with a shorter tear breakup time.^[15]^ Bacterial ulcers seem to be more advanced in thyroid eye disease patients; however, the relationship and mechanism has not yet been fully established.^[15]^ Confocal microscopy has revealed signs of subclinical diffuse corneal inflammation in active GO with increased stromal active keratocytes that release and are regulated by multiple inflammatory cytokines.[^16^,^17^] All of these factors may contribute to aggregation of the corneal ulcer.

Infection prevention is a critical part of intensive medical care. Consensus suggests that a mechanical eye cover with closed tracheal suction are ways to reduce respiratory pathogen colonization for patients with copious sputum, and if an open endotracheal suction system is used, the suction tube should never cross the patient's facial area during and after suctioning to avoid iatrogenic bacterial splash.[^1^,^13^,^19^] Eye hygiene is required to prevent ocular infection, and ointment or ocular discharge should be removed regularly with saline or sterile water-soaked gauze. As some evidence suggests normal saline can cause ocular surface drying with early tear evaporation, sterile water may be preferred for those with a preexisting corneal epithelium defect.^[19]^

Prophylactic usage of topical antibiotics on sedated patients is controversial, with the emergence of multi-resistant strains as the major concern. Furthermore, topical medications used in both eyes facilitate the accidental spread of pathogen transmission from one eye to another. In our experience, ointment also makes it more difficult for ICU nursing staff not fully trained in ocular assessment to detect corneal clouding or conjunctival discharge, which delays ophthalmological referrals. To the best of our knowledge, no large randomized study has been performed to evaluate its efficiency, yet topical antibiotics may have certain protective effects for those with a positive respiratory culture. Mela et al^[18]^ used a strict eye care protocol in 70 ventilated ICU patients and applied prophylactic topical antibiotics every 2 hours in those with positive conjunctival or tracheal cultures. In 7 patients with conjunctival bacterial colonization combined EK, none developed infectious keratitis.^[18]^ In our case, although gentamicin ointment was used twice daily, it had an insufficient bactericidal effect against *P aeruginosa*.

Treatment approaches for sedated ICU patients with GO and EK have several challenges because the ocular condition rapidly worsens and corticosteroids, the drug of choice in active GO, may have a negative impact on other systemic critical illness. Prevention and early recognition of corneal complications through ocular management is the key. Therefore, ophthalmologists and intensive unit staff could cooperate to create an efficient eye care protocol including careful risk assessment, regular lid position documentation, eyelid cleansing, and corneal protection with early ophthalmological consultation in suspicious infection cases to reduce serious visual impairments in these patients.

## 4 Conclusion

With improvements in intensive medical care and better survival prognoses, a dedicated, evidence-based eye care regimen is essential to decrease ocular complications in the ICU. Sedation, mechanical ventilation, and an open suction system are the red flags of ocular surface injury. GO also increases ocular surface inflammation and corneal exposure that should alert ICU staff and ophthalmologists of the high possibility of EK and corneal infection. With careful history taking, these cases should be better recognized and then treated and followed up aggressively.

## Acknowledgments

The authors would like to thank all colleagues who contributed to this paper and we would like to express our sincere thanks to anonymous reviewers and the editor for their comments.

## Author contributions

**Resources:** Yun-Chen Hsieh.

**Writing – original draft:** Yun-Chen Hsieh.

**Writing – review & editing:** Yun-Chen Hsieh, Chun-Chen Chen.
